# Promoting ER stress in a plasmacytoid dendritic cell line drives fibroblast activation

**DOI:** 10.1186/s12964-025-02057-7

**Published:** 2025-02-07

**Authors:** Beatriz H. Ferreira, Inês S. Silva, Andreia Mendes, Fátima Leite-Pinheiro, Adrienne W. Paton, James C. Paton, Iola F. Duarte, Philippe Pierre, Catarina R. Almeida

**Affiliations:** 1 Institute of Biomedicine (iBiMED), Department of Medical Sciences, University of Aveiro, Aveiro, Portugal; 2 CICECO - Aveiro Institute of Materials, Department of Chemistry, University of Aveiro, Aveiro, Portugal; 3 Department of Molecular and Biomedical Science, Research Centre for Infectious Diseases, University of Adelaide, Adelaide, Australia; 4 LAQV-REQUIMTE, Department of Chemistry, University of Aveiro, Aveiro, Portugal; 5 Centre d’Immunologie de Marseille-Luminy, Aix Marseille Université, CNRS, INSERM, Marseille Cedex 9, 13288 France; 6 Shanghai Institute of Immunology, Department of Microbiology and Immunology, Shanghai Jiao Tong University School of Medicine, Shanghai, 200025 China

**Keywords:** UPR, PERK, Cell-cell communication, Fibrosis, Plasmacytoid dendritic cells, Fibroblasts, ER stress, Scleroderma, Interstitial lung disease

## Abstract

**Background:**

Fibrosis remains a major complication in several chronic diseases, including systemic sclerosis (SSc). Plasmacytoid dendritic cells (pDCs) are innate immune cells that play a key role in the development of fibrosis in SSc patients, through still poorly defined mechanisms. Interestingly, endoplasmic reticulum (ER) stress signaling pathways are dysregulated in pDCs from patients with SSc, but their contribution to fibrosis remains unclear. Thus, this study aimed to unravel the mechanisms behind the involvement of pDCs and ER stress in fibrosis.

**Methods:**

To address this question, we established an in vitro model designed to study the interactions between pDCs and fibroblasts. More specifically, IMR-90 fibroblasts were co-cultured with CAL-1, a pDC cell line. ER stress was then induced by the bacterial toxin SubAB. Extracellular matrix (ECM) production was assessed using immunoblotting, qPCR and confocal microscopy. The importance of cell-to-cell contact was investigated using conditioned media (CM) and transwell assays.

**Results:**

Direct contact of CAL-1 and IMR-90 cells under ER stress conditions led to increased expression of fibronectin and alpha-smooth muscle actin (α-SMA). This effect required expression of the ER stress signaling sensor protein kinase R-like ER kinase (PERK) in pDCs and was observed only upon direct contact between both cell types.

**Conclusions:**

Overall, our data suggest that ER stress induction in pDCs promotes fibroblast activation, which may contribute to the development of fibrosis in SSc.

**Supplementary Information:**

The online version contains supplementary material available at 10.1186/s12964-025-02057-7.

## Background

Systemic sclerosis (SSc) patients commonly exhibit fibrosis of the skin, which can also extend to internal organs [1, 2]. Fibrosis results from excessive production of extracellular matrix (ECM) proteins, such as fibronectin and collagens, mainly by activated fibroblasts, leading to the disruption of tissue architecture, organ dysfunction and, eventually, organ failure [3]. Despite being a rare pathology, SSc has the highest mortality rate among rheumatic disorders, with lung involvement being the leading cause of death [4, 5]. Plasmacytoid dendritic cells (pDCs) are innate immune cells specialized in producing high amounts of type I interferons (IFN), which have been associated with SSc [6]. In fact, pDCs accumulate in the affected tissues of SSc patients and studies have shown that depleting pDCs in mouse models ameliorates fibrosis [7–11]. Moreover, activated pDCs contribute to the elevated levels of the type I IFN observed in SSc through the chronic production of IFN-α [8, 12, 13].

Different physiological conditions, particularly when there is a high demand for protein synthesis and folding, may promote endoplasmic reticulum (ER) stress. After accumulation of unfolded and misfolded proteins, immunoglobulin heavy-chain-binding protein (BiP) dissociates from the ER stress sensors – protein kinase R-like ER kinase (PERK), inositol-requiring enzyme 1 (IRE1) and activating transcription factor 6 (ATF6) – and the unfolded protein response (UPR) is initiated to favour ER homeostasis. The UPR may be induced upon environmental alterations, such as nutrient deprivation, hypoxia, heat, or infection. The subtilase cytotoxin SubAB is an AB5 toxin produced by a Shiga toxin-producing *Escherichia coli* (STEC) strain that induces ER stress by cleaving BiP [14]. Promoting ER stress in human lung fibroblasts with pharmacological inducers upregulates the production of collagens and alpha-smooth muscle actin (α-SMA), a fibroblast activation marker [15–17]. However, the impact of more natural and targeted ER stress inducers, such as SubAB, on fibrosis induction has not been studied so far.

ER stress markers have been reported to be upregulated in SSc and importantly, dysregulation of ER stress signaling pathways has been observed in circulating pDCs isolated from SSc patients [13, 15]. Even though recent studies have linked pDCs, ER stress, and SSc, the exact mechanisms underlying their role in fibrosis remain unclear. Given that pDCs and fibroblasts are likely to interact in affected tissues, this study explores the effect of ER stress on co-cultures of pDCs and fibroblasts.

## Methods

### Cell lines

CAL-1 (a kind gift from Dr. Takahiro Maeda, Nagasaki University, Japan) and IMR-90 cell lines (CCL-186™, ATCC, Manassas, VA, USA and I90-15, Coriell Institute for Medical Research, Camden, NJ, USA) were maintained in RPMI 1640 medium with 2 mM L-glutamine (Gibco, Thermo Fisher Scientific, Waltham, MA, USA), supplemented with 10% heat-inactivated foetal bovine serum (FBS; Sigma-Aldrich, St. Louis, MO, USA), 1x non-essential amino acids and 1 mM sodium pyruvate (both from Gibco). Cells were cultured in a humified atmosphere at 37 °C with 5% CO_2_ and were regularly tested for mycoplasma contamination. Both CAL-1 and IMR-90 cells were used bellow passage 15.

### Reagents

SubAB and the inactive form SubA_A272_B were produced as previously described [14]. Tunicamycin (Tm) (cat#SML1287) and ascorbic acid (cat#A4544) were purchased from Sigma-Aldrich. CL307 (cat#TLRL-C307) was from InvivoGen (San Diego, CA, USA). HA15 was a kind gift from Dr. Stéphane Rocchi [18].

### Co-cultures and transwell assays

IMR-90 cells were detached using 0.25% trypsin-EDTA (Gibco), resuspended in growth medium and 200,000 or 90,000 cells were plated in 6 or 12-well plates, respectively. Cells were incubated overnight, prior to 24 h starvation with RPMI 1% FBS, 1x non-essential amino acids and 1 mM sodium pyruvate. CAL-1 cells were then added to IMR-90 at a 1:10 ratio (IMR-90 and CAL-1, respectively), and cells were further incubated in fresh culture medium supplemented with ascorbic acid (50 µg/mL). Cells were exposed to SubAB (1 µg/mL), SubA_A272_B (1 µg/mL), CL307 (1 µM), Tm (1 µM) or HA15 (7.5 µM) for 24 h to 72 h. The same approach was used for transwell experiments (6-well plates with 0.4 μm polyester membrane; Costar, Washington DC, USA), but here IMR-90 and CAL-1 or IMR-90 – CAL-1direct co-cultures were seeded in the bottom and upper chamber, respectively, at the same ratio as described above. Cells treated for 24 h, 48 h and 72 h were used for qPCR, immunocytochemistry or immunoblotting, respectively.

### Conditioned media (CM) experiments

After monoculture and co-culture (24 h incubation), CM were collected, centrifuged for 5 min at 300 x *g* and 4 °C to remove cell debris, aliquoted and stored at -80 °C for later use. On the day of the experiment, CM were thawed and added to IMR-90 at a 2x dilution in RPMI medium. Cells were incubated with the CM for 24 h to 72 h. RPMI media alone incubated for 24 h in the same conditions was used as control.

### Immunoblotting

Lysis of the cells was performed with RIPA buffer (25 mM Tris-HCl pH 7.6, 150 mM NaCl, 1% NP-40; 1% sodium deoxycholate, 0.1% SDS) supplemented with Pierce Protease Inhibitor Tablets (Thermo Fisher Scientific), sodium fluoride (NaF 50 mM; Sigma-Aldrich) and sodium orthovanadate (Na_3_VO_4_ 0.2 mM; Sigma-Aldrich), two phosphatase inhibitors, and MG132 (10 mM) a proteasome inhibitor. After centrifugation (14000 rpm, 20 min, 4 °C), the supernatant was collected, and protein quantification was determined using the bicinchoninic acid (BCA) protein assay kit (Pierce, Thermo Fisher Scientific). 10 µg of soluble proteins were run in 8% polyacrylamide gels, alongside with NZYColour Protein Marker II (NZYTech, Lisbon, PT), and transferred to Immobilon^®^-P PVDF membranes 0.45 μm pore size (Millipore, Burlington, MA, USA).

Membranes were blocked with 5% bovine serum albumin (BSA; NZYTech) in tris-buffered saline with 0.05% Tween 20 (Sigma-Aldrich; TBS-T) for 1 h, and primary antibodies were incubated overnight at 4 °C. Anti-PERK (cat#C33E10, 3192), anti-phospho-eukaryotic translation factor subunit alpha (p-eIF2α; cat#3597), anti-eIF2α (cat#9722) and anti-STING (cat#13647) were from Cell Signaling Technology (Danvers, MA, USA) and were used at a 1:1000 concentration. Anti-growth arrest and DNA damage-inducible protein (GADD34; cat#10449-1-AP; 1:1000) was from Proteintech (Rosemont, IL, USA) and anti-alpha-smooth muscle actin (α-SMA; cat#14-9760-80; 1:1000) was from Invitrogen (Grand Island, NY, USA). Anti-β-actin (cat#A5441; 1:50000) was from Sigma-Aldrich. Anti-human fibronectin HFN 7.1 (RRID: AB_528244) culture supernatant [0.5 µg/mL, Developmental Studies Hybridoma Bank (DSHB), Iowa City, IA, USA] was used. HFN 7.1 was deposited to the DSHB by Klebe, R.J. (DSHB Hybridoma Product HFN 7.1). Secondary antibodies HRP-linked were incubated for 1 h at room temperature (RT). Anti-mouse (cat#715-035-151, 1:10000) was purchased from Jackson ImmunoResearch (West Grove, PA, USA) and anti-rabbit (cat#7073, 1:5000) was from Cell Signaling Technology. When necessary, membrane stripping was performed with Restore™ Western Blot Stripping Buffer (Thermo Fisher Scientific) for 20 min at 37 °C. All membrane washes were performed with TBS-T for 10 min, three at a row. Blots were developed using chemiluminescence (ECL Plus or ECL Select; Pierce and Cytiva, Marlborough, MA, USA, respectively) accordingly to the manufacturer’s protocol in a ChemiDoc Imaging System (Bio-Rad, Hercules, CA, USA). Quantification was performed using Image Lab 6.1 software (Bio-Rad) and data was normalized to β-actin or total protein calculated from the entire blot to ensure an accurate assessment (stained with Coomassie Brilliant Blue R-250; Bio-Rad).

### Immunocytochemistry and microscopy analysis

IMR-90 cells and IMR-90 – CAL-1 direct co-cultures previously seeded in 12-mm glass coverslips were treated with SubAB for 48 h for fibronectin and α-SMA staining. For controls with CAL-1 cells alone, after treatment, cells were harvested and seeded on 12-mm Alcian blue-pretreated coverslips for 10 min at 37 °C. Cells were fixed for 15 min with 4% paraformaldehyde (PFA; Alfa Aesar) in phosphate-buffered saline (PBS) and permeabilized with 0.2% Triton X-100 (Sigma-Aldrich) for 10 min at RT. Following blocking with 1% BSA at RT for 10 min, cells were incubated overnight at 4 °C with the primary antibody against non-conjugated fibronectin (HFN 7.1, DSHB, 2 µg/mL) and for 1 h at RT with the secondary antibody (Alexa Fluor™ 647, cat#A21263, Invitrogen). After incubation with each antibody, three washes with PBS were performed. For F-actin staining, after treatments for 1 h and cell fixation, permeabilization was performed with 0.5% Triton X-100. Cells were then incubated for 20 min with 5 U/mL CF^®^ 488 A Phalloidin Conjugate (cat#00042-T, Biotium, Fremont, CA, USA) and washed 3 times with PBS. Nuclear staining was performed with DAPI (300 nM; Invitrogen) for 4 min. Slides were mounted using ProLong Gold Antifade (Thermo Fisher Scientific) and incubated 24 h at RT in the dark.

Confocal images were acquired using a Zeiss LSM 880 confocal microscope (Zeiss, Oberkochen, DE). Fibronectin was observed under a Plan-Apochromat objective (20x magnification), and for F-actin an Alfa Plan Apochromat objective (100x magnification) was used. Images were processed using ZEN Black and ZEN Blue software (Zeiss).

Measurements of fluorescence intensity for fibronectin were performed using the Fiji Image J2 software (NIH, Bethesda, MD, USA). The background was taken in consideration in the analysis and the mean of at least 4 images per biological replicate was used.

### Quantitative reverse transcription polymerase chain reaction (RT-qPCR)

Total RNA extraction was performed using the NZY Total RNA isolation kit (NZYTech), including a DNA digestion step with RNase-free DNase (NZYTech), accordingly to manufacturer’s instructions, and quantified by NanoDrop (DS-11 spectrophomoter, DeNovix Inc, Wilmington, DE, USA). cDNA was synthesized from 500 ng of RNA using the Superscript II Reverse Transcriptase (Invitrogen), dNTPs NZYMix (NZYTech) and random hexamers (Thermo Scientific), following manufacturer’s instructions.

The primer sequences used for fibronectin were 5’-TGTCAGTCAAAGCAAGCCCG-3’ and 5’-TTAGGACGCTCATAAGTGTCACCC-3’; for α-SMA were 5’-GTGAAGAAGAGGACAGCACTG-3’ and 5’-CCCATTCCCACCATCACC-3’; for type 1 collagen were 5’-CTGTAAACTCCCTCCATCCC-3’ and 5’- GTCCATGTGAAATTGTCTCCC-3’; for GAPDH were 5’- TCGGAGTCAACGGATTTGGT-3’ and 5’- TTCCCGTTCTCAGCCTTGAC-3’; and for transforming growth factor (TGF)-β1 were 5’-GGAAATTGAGGGCTTTCGCC-3’ and 5’-CCGGTAGTGAACCCGTTGAT-3’ (Invitrogen). GAPDH was used as reference gene. Quantitative PCR amplification was performed in duplicate using 2x SYBR Green qPCR Master Mix (Low Rox; Selleckchem, Houston, TX, USA) using 10x diluted cDNA and 300 nM of each specific primer on a 7500 Real Time PCR System or QuantStudio™ 3 Real-Time PCR System (Applied Biosystems, Waltham, MA, USA). Data analysis was performed using the quantitative 2^−ΔΔCT^ method.

### Cytokine quantification by enzyme-linked immunosorbent assay (ELISA)

Human TGF-β1 was quantified in cell culture media using Human TGF-beta 1 DuoSet ELISA (cat#DY240, Bio-Techne, R&D Systems, Minneapolis, MN, USA) following manufacturer’s instructions.

### Statistical analysis

Statistical analyses were performed using GraphPad Prism 9.0.0 software (GraphPad Software, Inc., La Jolla, CA, USA). Each dot indicates an independent experiment and data are presented as mean ± standard deviation (SD). Normality was assessed using Shapiro-Wilk test and differences in variances was tested using Brown-Forsythe test. Comparisons between groups were performed as mentioned in the figure legends. *p ≤ 0.5, **p ≤ 0.01, ***p ≤ 0.001, ****p ≤ 0.0001.

## Results

### Exposure of IMR-90 – CAL-1 co-cultures to SubAB promotes fibronectin and α-SMA expression

**Fig. 1:**
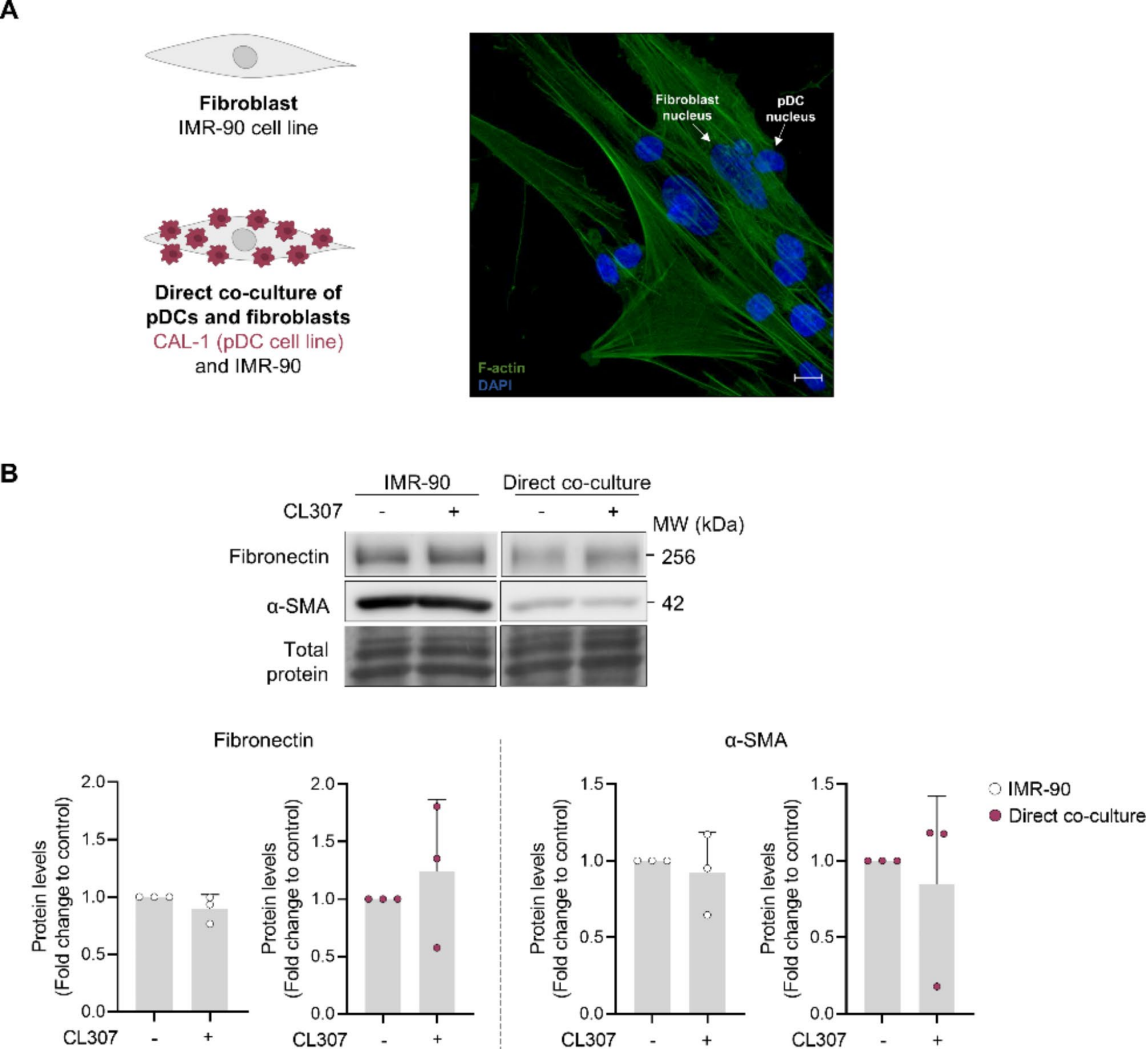
IMR-90 and CAL-1 interact in co-culture. **(A)** IMR-90 cells were cultured alone or in combination with CAL-1 cells (1:10 ratio, respectively), and F-actin (green) was observed by confocal microscopy after 1 h of co-culture. CAL-1 were distinguished from IMR-90 cells due to the different sizes in the cells and shape of the nuclei (blue). Scale bar = 10 μm. Representative images of 2 biological replicates. **(B)** Fibronectin and α-SMA protein levels were analysed by immunoblot in IMR-90 monoculture and CAL-1 – IMR-90 co-culture exposed to CL307 (1 µM) for 72 h. Data represents mean ± SD and each dot represents a biological replicate. Student’s *t* test with Welch’s correction

To study the impact of pDCs on fibroblast behaviour, we established an in vitro co-culture model with CAL-1 cells, a pDC cell line, and IMR-90 lung fibroblasts at a 10:1 ratio, respectively (Fig. 1A). CAL-1 cells recapitulate the phenotype of human primary pDCs, providing a powerful tool to study an immune cell population which is otherwise difficult to investigate in detail due to its scarcity [19, 20]. CAL-1 cells were observed forming conjugates with the fibroblasts, which did not come off easily with several washing steps (Fig. 1A and Fig. S1, Additional file 1). When we compared the amount of different ECM components in the co-culture with a control comprising a combination of separately cultured IMR-90 and CAL-1 cell pellets, we observed a decrease in both protein and mRNA levels of α-SMA, as well as a reduction in type I collagen mRNA levels (Fig. S2A, B, Additional file 1). This may be due to a possible difference in growth rates between CAL-1 and IMR-90 cells in the co-culture, potentially affecting the cell:cell ratio, with a decrease in the proportion of cells producing α-SMA. To test whether pDC activation may affect fibroblast activation, we also treated IMR-90 – CAL-1 co-cultures with the Toll-like receptor 7 (TLR7) agonist CL307, which promotes IFN-β secretion by CAL-1 cells (data not shown). Neither IMR-90 monoculture nor IMR-90 – CAL-1 co-cultures exhibited altered expression of fibronectin or α-SMA after treatment with CL307 (Fig. 1B).

**Fig. 2:**
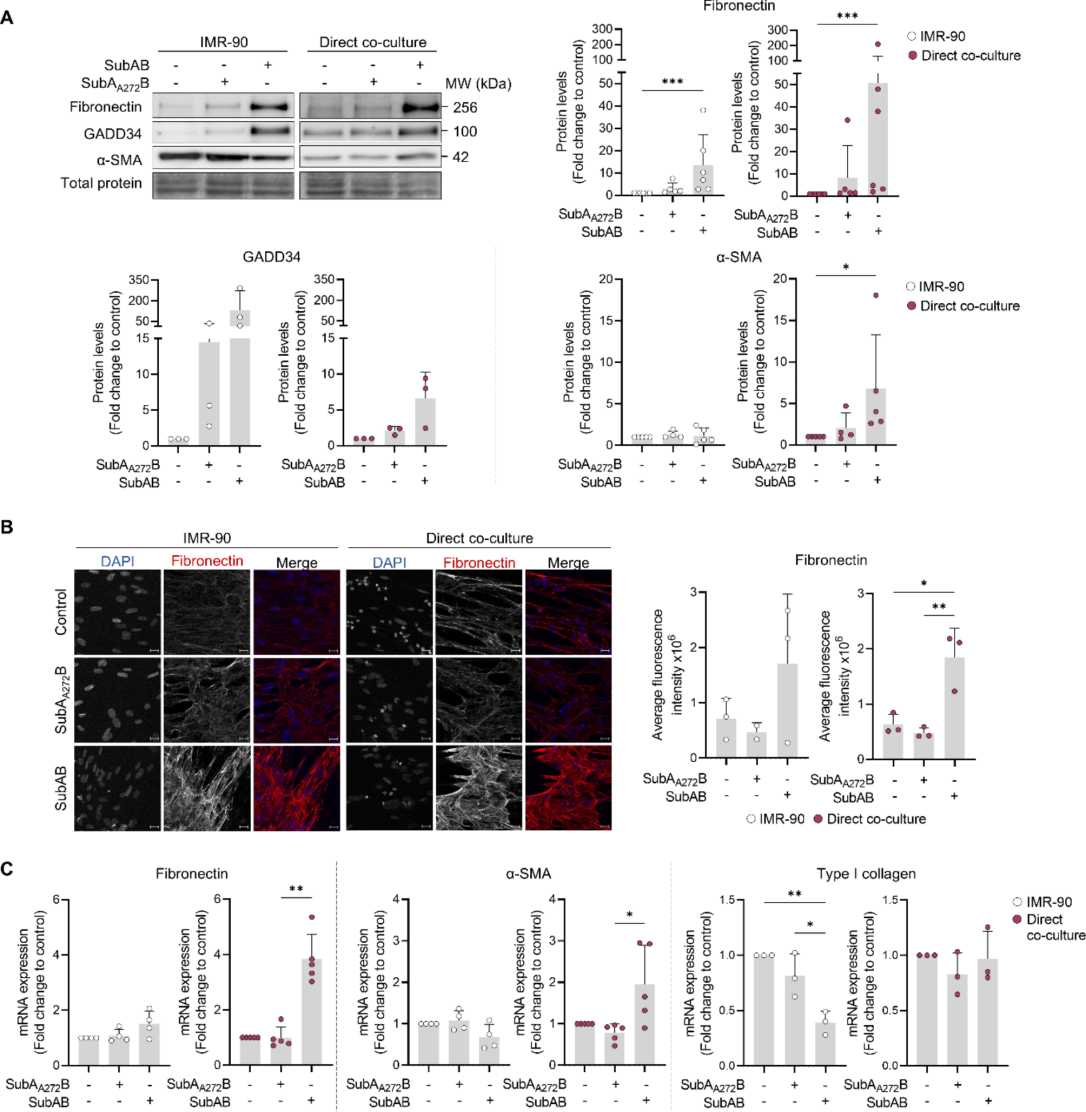
CAL-1 cells potentiate fibronectin and α-SMA production by SubAB-exposed IMR-90. IMR-90 cells were cultured alone or combined with CAL-1 cells and treated with SubAB or SubA_A272_B (1 µg/mL). Expression of fibronectin and α-SMA was analyzed by **(A)** immunoblot (72 h incubation, representative blot of at least 3 biological replicates), **(B)** confocal microscopy (48 h incubation, scale bar = 20 µM, representative images of 3 biological replicates) and **(C)** qPCR (24 h incubation). In **(A)** and **(C)** GADD34 protein levels and type I collagen mRNA expression, respectively, are also shown. Data represents mean ± SD and each dot represents a biological replicate. Kruskal-Wallis test followed by Dunnet’s multiple comparisons test (A – fibronectin and α-SMA direct co-culture; C – fibronectin direct co-culture) and one-way ANOVA followed by Sidak’s multiple comparisons test (A – GADD34 and α-SMA IMR-90; B; C – fibronectin IMR-90, α-SMA and type I collagen)

To test the impact of ER stress in the co-cultures, cells were then treated with SubAB. As expected, SubAB treatment induced UPR activation in both IMR90 cells alone and IMR-90 – CAL-1 co-cultures, as confirmed by the increased levels of GADD34 (Fig. 2A). GADD34 is produced downstream of the activated ER stress sensor PERK, and acts as a negative feedback protein, to attenuate UPR signaling. Incubation with a mutant form of SubAB, SubA_A272_B, which lacks the protease activity against BiP, did not trigger GADD34 expression. Treatment of IMR-90 fibroblasts monoculture with SubAB led to an increase in fibronectin protein levels, without affecting α-SMA, and to a decrease in type I collagen mRNA levels (Fig. 2). CAL-1 cells alone did not express fibronectin, α-SMA, nor type I collagen (Fig. S2C-E, Additional file 1). However, most importantly, adding SubAB to the IMR-90 – CAL-1 co-cultures promoted the production of fibronectin and α-SMA at both protein and mRNA levels, an effect not observed with the control SubA_A272_B (Fig. 2).

Thus, SubAB-induced ER stress, in combination with the presence of CAL-1 cells, promotes fibronectin production and fibroblast activation, two hallmarks of fibrosis.

### Fibroblast activation in IMR-90 – CAL-1 co-cultures requires PERK expression on CAL-1

**Fig. 3:**
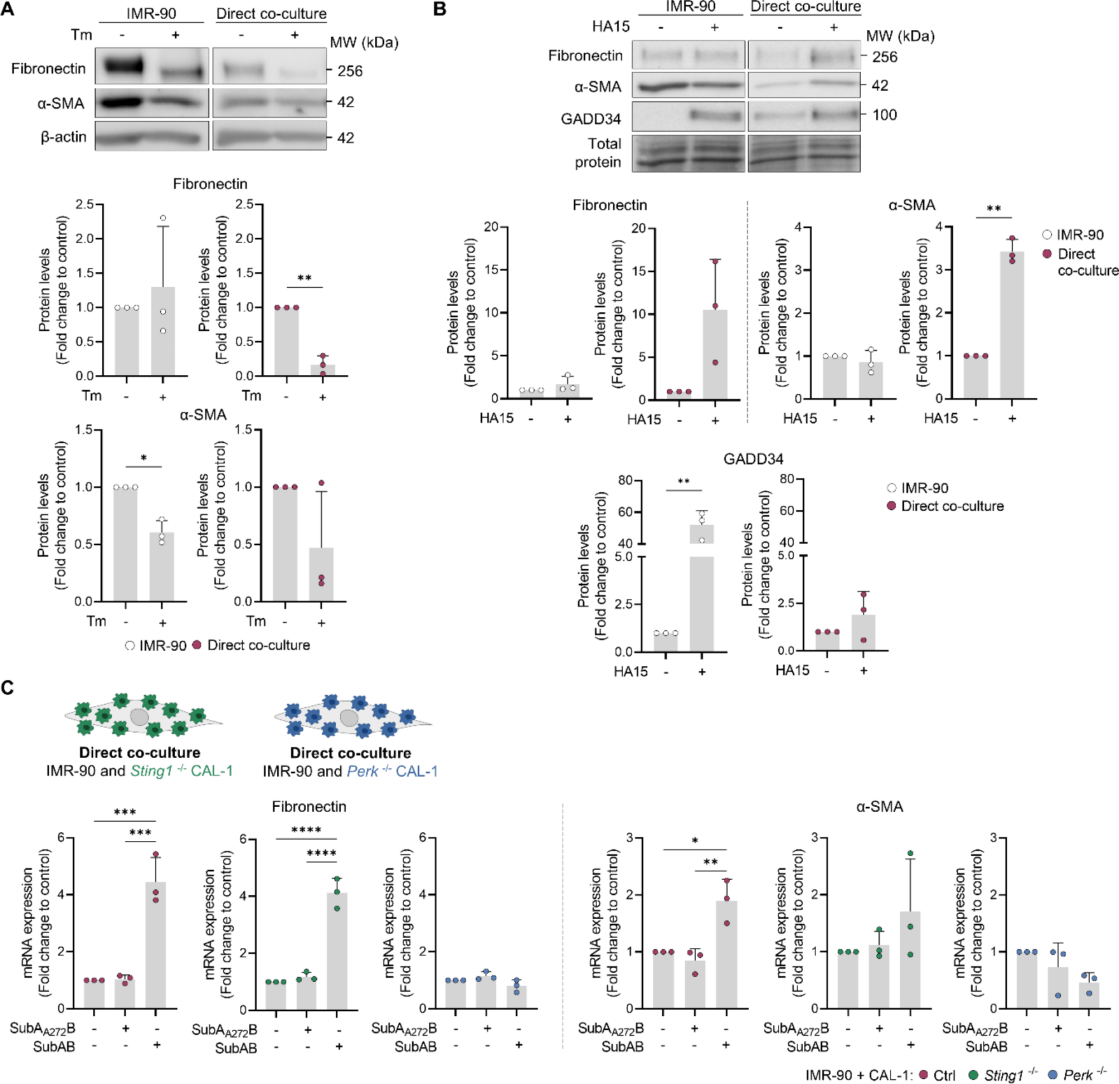
BiP inhibition/depletion and PERK activation affect fibronectin and α-SMA expression in IMR-90–CAL-1 co-culture. Fibronectin, α-SMA and/or GADD34 protein levels were analysed by immunoblot in IMR-90 alone or in co-culture with CAL-1 and treated with **(A)** tunicamycin (Tm; 1 µM) or **(B)** HA15 (7.5 µM) for 72 h. **(C)** Levels of fibronectin and α-SMA mRNA were determined in the direct co-culture of IMR-90 and control (Ctrl), *Sting1*^−/−^ or *Perk*^−/−^ CAL-1 cells exposed to SubAB for 24 h. Data represents mean ± SD and each dot represents a biological replicate. Blots are representative of 3 biological replicates. Statistical analysis was performed using unpaired t test with Welch’s correction **(A, B)** or one-way ANOVA followed by Sidak’s multiple comparisons test **(C)**

The effect of other ER stress inducers, namely Tm, which inhibits protein glycosylation, and HA15, a BiP inhibitor, was also tested. Unexpectedly, Tm treatment induced a reduction in fibronectin and α-SMA protein levels in the co-culture and IMR-90 monoculture, respectively (Fig. 3A). ER stress induction in IMR-90 and CAL-1 cells was confirmed by analysing PERK and eIF2α phosphorylation, as well as GAD34 upregulation, after 6 h of treatment with Tm (Fig. S3, Additional file 1). On the other hand, HA15 affected the direct co-culture in a way more reminiscent of SubAB, upregulating fibronectin and α-SMA protein levels (Fig. 3B). However, HA15 did not promote accumulation of fibronectin in monocultured IMR-90 cells, although GADD34 was upregulated even after 3 days of incubation (Fig. 3B).

We have previously observed that pDC exposure to SubAB triggers activation of PERK and the stimulator of IFN genes (STING), which are key for the induced pDC activation [21]. Therefore, to clarify the role of PERK and STING expression by CAL-1 cells, we directly co-cultured IMR-90 with either *Sting1*^*−/−*^*or Perk/EIF2AK3*^−/−^ CAL-1 cells (Fig. 3C and Fig. S4, Additional file 1) [21]. While the absence of STING did not impact fibronectin expression, PERK depletion in CAL-1 cells abrogated the upregulation of fibronectin and α-SMA expression in the SubAB-treated co-culture (Fig. 3C).

Together, these results suggest that BiP inhibition/degradation and the consequent induction of UPR in CAL-1 cells, specifically the PERK pathway, promotes the production of fibronectin and α-SMA in the CAL-1 and IMR-90 co-culture.

### Fibroblast activation in the presence of CAL-1 cells requires intercellular contact

**Fig. 4:**
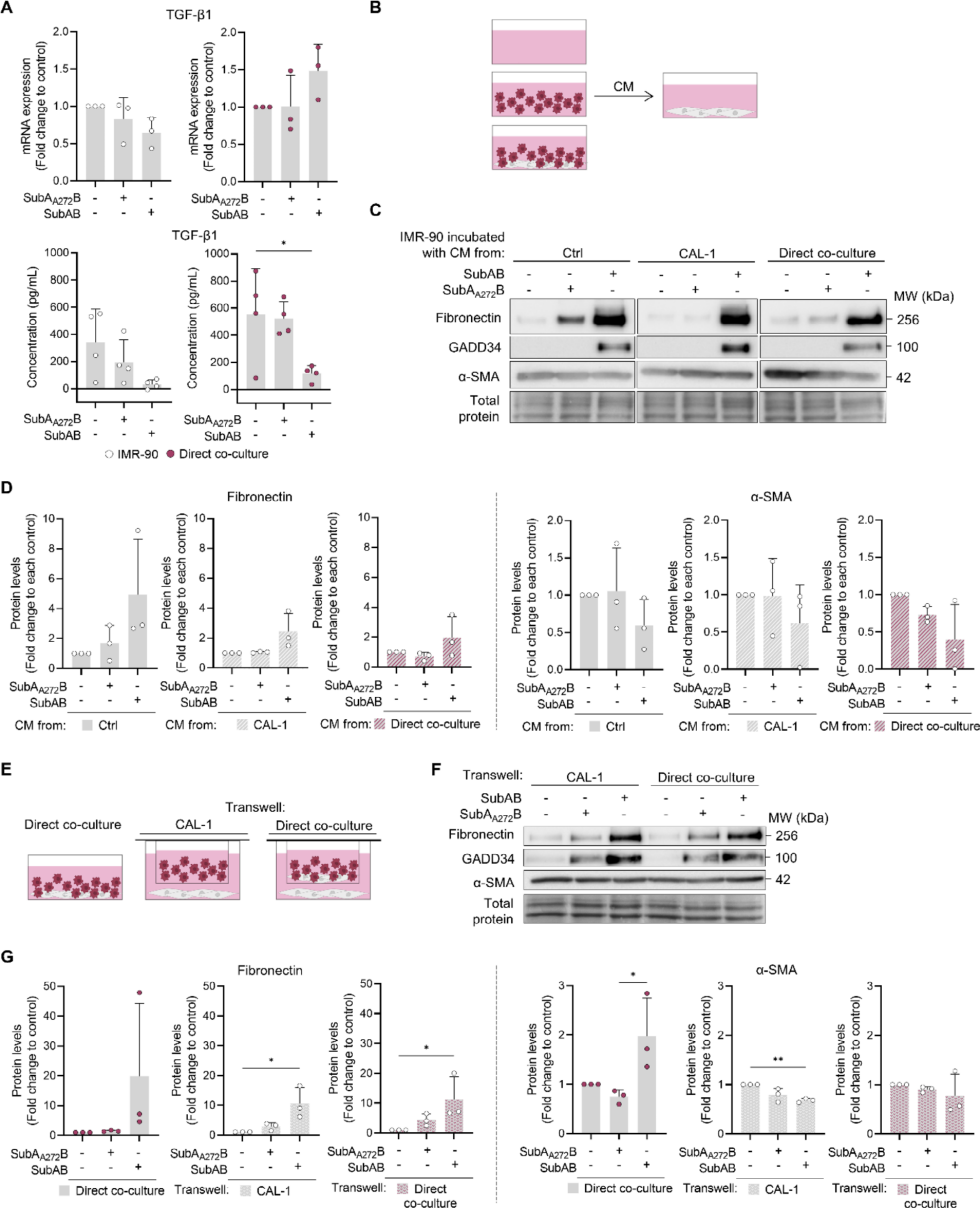
IMR-90 activation induced by CAL-1 during SubAB-induced ER stress is cell-to-cell contact dependent. **(A)** TGF-β1 mRNA and secretion levels analysed by qPCR and ELISA, respectively, in the direct co-culture exposed to SubAB for 24 h. IMR-90 were **(B-D)** incubated with conditioned media (CM) from either CAL-1 or from direct co-cultures. Media without cells was used as control (ctrl); or **(E-G)** cultured in transwells, separated from CAL-1 or direct co-cultures in SubAB-induced ER stress conditions. Direct co-culture was used as control. **(C**,** D**,** F**,** G)** Fibronectin, GADD34 and α-SMA protein levels were analyzed by immunoblot. Data represents mean ± SD and each dot represents a biological replicate. Statistical significance was evaluated using one-way ANOVA followed by Sidak’s multiple comparisons test, except for fibronectin levels of IMR-90 indirectly co-cultured with the direct co-culture **(G)** where Kruskal-Wallis followed by Dunnet’s multiple comparisons test was used

TGF-β1 is a well-known profibrotic factor that directly activates fibroblasts via direct binding to TGF-β receptor 2, inducing ECM production, and can also modulate immune cell function [22]. To elucidate if the SubAB-induced effect is accompanied by TGF-β1 upregulation, we analysed its mRNA expression and protein levels in the cell culture media. We found no induction of TGF-β1 expression by SubAB, while its secreted levels were reduced, possibly due to protein synthesis inhibition downstream of PERK activation (Fig. 4A). Thus, fibroblast activation in the SubAB-treated co-cultures is not driven by TGF-β1.

To test if fibroblast activation can be mediated by other factors secreted by cells, IMR-90 fibroblasts were incubated with CM from CAL-1 or IMR-90 – CAL-1 co-culture (Fig. 4B). CM was generated from non-treated cells and cells exposed to SubA_A272_B or SubAB. As a control, acellular medium was incubated under the same conditions. We observed that CM from either CAL-1 or the direct co-culture treated with SubAB did not promote an increase in the production of fibronectin or α-SMA by IMR-90 in relation to each respective control (Fig. 4C, D). Furthermore, IMR-90 fibroblasts were co-cultured in the lower compartment of transwells, while CAL-1 cells or IMR-90 – CAL-1 co-culture were placed in the upper compartment (Fig. 4E), in the presence or absence of SubAB. Neither of these conditions led to an increase in α-SMA production by IMR-90 (Fig. 4F, G). A slight increase in fibronectin protein levels was observed in SubAB conditions, more likely from the effect of SubAB that was added to the culture (directly in the transwells or during the generation of CM), which is corroborated by the presence of GADD34 (Fig. 4C, F). These results suggest that the soluble factors secreted by SubAB-treated CAL-1 cells, either in monoculture or in contact with IMR-90 fibroblasts, are not sufficient to promote fibroblast activation.

Collectively, these findings suggest that ER stress leads CAL-1 to promote activation of IMR-90 fibroblasts in a cell-to-cell contact-dependent manner.

## Discussion

ER stress can be triggered under conditions of high protein synthesis demand, such as fibrosis and inflammation. Indeed, upregulation of ER stress pathways has been described in peripheral blood mononuclear cells (PBMCs) from SSc patients [23]. It has also been reported that pDCs from the lungs of these patients exhibit upregulated stress pathways [10], and pDCs isolated from the blood of SSc patients show dysregulation of ER stress signaling pathways [13]. Here, we show that SubAB, an ER stress inducer, promotes expression of fibronectin and α-SMA in co-cultures of fibroblast and a pDC cell line, in a PERK- and cell-to-cell contact-dependent manner. Even though pDCs primarily circulate through lymphoid organs, they are recruited to tissues during inflammation. In fact, pDC numbers are higher in the skin and lungs of SSc patients, while being reduced in the blood [7, 8, 10]. Once in the affected tissues, pDCs can directly contact and influence fibroblasts, one of the main producers of ECM proteins. Moreover, pDCs can produce high levels of type I IFN, thus contributing to the signature found in SSc patients. The demand for production of high amounts of type I IFN and other factors may surpass the maximum capacity of the ER for protein synthesis and folding, leading to ER stress. Also, exposure to environmental factors, including occupational exposures and toxins, which is also a risk factor for developing lung fibrosis, can cause cellular stress [24]. Importantly, ER stress has been implicated in many fibrotic disorders [25], and there is also evidence for its involvement in SSc [13, 23, 26].

Under ER stress conditions, BiP dissociates from the ER stress sensors, resulting in initiation of the UPR. In this work, we primarily used the subtilase cytotoxin SubAB, which directly cleaves BiP, while also testing other ER stress inducers. HA15 revealed a similar effect to SubAB, increasing fibronectin and α-SMA expression in the direct IMR-90 – CAL-1 co-culture. Both SubAB and HA15 target BiP, even though through different mechanisms, with HA15 inhibiting the ATPase activity of BiP and consequently perpetuating the accumulation of misfolded proteins. On the other hand, Tm, which was found not to have the same effect as SubAB and HA15 in the direct co-cultures, inhibits protein glycosylation, resulting in the accumulation of unfolded proteins. Inhibition of protein glycosylation can impair the production of proteins that need to undergo this post-translation modification, such as ECM proteins, as well as cytokines or other factors that may mediate the effects of SubAB-treated CAL-1 on IMR-90 cells [27]. Moreover, it can alter glycosylation of cell surface proteins, which may influence the interaction between both cell types [27]. Others have described that Tm induces α-SMA and collagen production by fibroblasts [15, 16]. Conversely, in chondrocytes, the same ER stress inducer inhibited collagen synthesis [28]. All these studies were performed using different cells, which can explain the discrepant results. Despite the varying responses of the direct co-culture to the tested ER stress inducers, we confirmed that SubAB-induced fibronectin and α-SMA production is directly linked to UPR activation in CAL-1 cells. Indeed, the direct co-culture of IMR-90 and *Perk*^−/−^ CAL-1 cells abrogated the fibrotic phenotype induced by SubAB.

ER stress can activate immune cells and stimulate the production of inflammatory mediators [29], and we have previously observed that SubAB induces type I IFN expression by pDCs [21]. Also, SSc patients display a type I IFN signature to which pDCs are important contributors [8, 12]. Nonetheless, the fibroblast activation observed in the direct co-culture was not solely dependent on type I IFN, as the induction of type I IFN by TLR7 activation was not sufficient to promote fibroblast activation. However, it remains possible that, in vivo, these cytokines affect cells other than fibroblasts and may also contribute to fibroblast activation. We also considered whether fibroblast activation could be mediated by TGF-β1, a well-known pro-fibrotic factor that directly activates fibroblasts [22]. However, there was no increase in its expression, suggesting that TGF-β1 is not responsible for this effect.

Other cytokines, such as CXCL4, IL-11 and IL-6 have also been described as pro-fibrotic, being capable of inducing fibroblast activation [11, 30–33]. Nevertheless, experiments with CM and transwells clarified that fibroblast activation mediated by SubAB-treated CAL-1 cells is not merely due to soluble factors released by the cells, as the effect was lost in experiments using CM or transwells. But while soluble cytokines alone may not be sufficient for the effects described in this study, they may act indirectly, by promoting cellular interactions. For example, cytokines may impact on expression of surface mediators such as cell adhesion molecules or connexins, or contribute to the amplification of signalling networks.

The increase in fibronectin and α-SMA expression was lost when IMR-90 were directly co-cultured with *Perk*^−/−^ cells, indicating that the effect reported here is dependent on PERK activation in CAL-1 cells. Thus, PERK activation may induce alterations in CAL-1 cells that facilitate fibroblast activation through direct contact. Some studies have shown that under certain conditions PERK can mediate calreticulin exposure at the cell surface [34–36]. Interestingly, pDc are known to express Notch ligand when activated [37] and calreticulin has been associated with Notch signalling, in the context of cardiac fibrosis [38]. Therefore, it can be hypothesized that PERK activation in CAL-1 cells leads to calreticulin exposure, which in turn drives Notch signalling, contributing to the observed upregulation of fibronectin and α-SMA expression by IMR-90. Deciphering the exact mechanism and signalling networks by which direct contact between fibroblasts and CAL-1 promotes fibroblast activation remains to be elucidated and will require extensive work in future studies.

Besides being pro-fibrotic and a biomarker for SSc, CXCL4 has been found to reduce the expression of UPR genes from the IRE1-XBP1 pathway in pDCs from SSc patients, while also promoting expression of type I IFN by activated pDCs, particularly in conditions of ER stress [13]. More recent work has also suggested that CXCL4 promotes pDC activation in fibrotic conditions by overcoming inhibition of type I IFN induced by the increased tissue stiffness that can be found in these tissues [39]. Our data suggests that soluble CXCL4 is not essential for fibroblast activation upon ER stress stimulation in the co-cultures. However, it remains unknown whether increasing concentrations of CXCL4, such as the ones that can be found at sites of tissue injury, affect pDC – fibroblast interactions in the presence of SubAB, contributing to production of even further amounts of ECM proteins.

Senescent cells, which have permanent cell-cycle arrest, can present a cellular senescence-associated secretory phenotype (SASP), which contributes to inflammation and fibrosis through the secretion of cytokines, chemokines and ECM [40]. Cellular senescence is associated with fibrosis and SSc [41, 42], and the UPR has been suggested to drive senescence [43]. Therefore, our observations may be related with this phenotype. Prolonged ER stress induced by SubAB triggers cell death in certain cell types and is lethal for mice [44–49]. Here, there was also visible cell death for timepoints longer than 24 h on both IMR-90 and CAL-1 cells (data not shown). Despite the occurrence of cell death, accumulation of ECM proteins can persist and contribute to tissue stiffness and, consequently, fibrosis. In SSc, endothelial cell apoptosis occurs even at early stages of the disease [50]. While some authors have reported resistance to apoptosis in SSc-derived fibroblasts and T lymphocytes [51–54], others found an increase in apoptotic cell numbers in tissues from SSc patients, including nonendothelial cells [55]. Senescent cells are usually characterized by resistance to apoptosis. Under our experimental conditions, it is possible that SASP contributed to ECM production at earlier timepoints. However, as time progressed and cells became unable to resolve ER stress, this culminated in cell death, a hypothesis that remains to be clarified.

Due to the limited number of primary pDCs that can be obtained from patients’ blood, this study was performed with a pDC cell line that closely recapitulates the pDC phenotype. However, as with other cell lines, CAL-1 cells do not fully reflect pDC biology and function [20]. And thus, while this work shows that a pDC cell line may interact with fibroblasts and contribute to their activation, it will be very important to confirm these observations with primary cells. The inability to separate pDCs and fibroblasts after direct co-culture also poses some limitations in the analysis of results, as all analyses, including those of intracellular protein and mRNA expression, were performed with both cell types present. We confirmed that CAL-1 cells do not express fibronectin and α-SMA, but alterations in the cell-to-cell ratio during co-culture, may influence the results. For example, direct contact between IMR-90 and CAL-1 cells led to a decrease in fibronectin and α-SMA levels, which could be attributed to variations in the cell-to-cell ratio. On the other hand, the upregulation of fibronectin and α-SMA levels induced by SubAB in direct pDC-fibroblast co-cultures was clear and confirmed through various techniques, making it unlikely that these observations are due to artefacts.

## Conclusion

Overall, we found that ER stress-activated CAL-1 cells could trigger ECM production and IMR-90 fibroblast activation through direct contact with fibroblasts, one of the central key players in fibrosis. Therefore, this work unveils a mechanism by which pDCs may contribute to fibrosis, contributing to the understanding of fibrotic processes and opening new perspectives for the development of innovative therapeutic approaches for fibrotic diseases, particularly for SSc, where pDCs are known to play a key role.

## Electronic supplementary material

Below is the link to the electronic supplementary material.

Supplementary Material 1

Supplementary Material 2

## Data Availability

The datasets used and/or analysed during the current study are available from the corresponding author on reasonable request.

## Abbreviations

ATF6: Activating transcription factor 6
BCA: Bicinchoninic acid
BiP: Immunoglobulin heavy-chain-binding protein
BSA: Bovine serum albumin
CM: Conditioned media
ECM: Extracellular matrix proteins
eIF2α: Eukaryotic translation factor subunit alpha
ER: Endoplasmic reticulum
GADD34: Anti-growth arrest and DNA damage-inducible protein
IFN: Interferon
ILD: Interstitial lung disease
IRE1: Inositol-requiring enzyme 1
PBMC: Peripheral blood mononuclear cell
PBS: Phosphate-buffered saline
pDC: Plasmacytoid dendritic cell
PERK: Protein kinase R-like ER kinase
PFA: Paraformaldehyde
RT: Room temperature
SASP: Senescence-associated secretory phenotype
SD: Standard deviation
SSc: Systemic sclerosis
STEC: Shiga toxin-producing *Escherichia coli*
STING: Stimulator of interferon genes
Tg: Thapsigargin
TGF-β: Transforming growth factor beta
TLR7: Toll-like receptor 7
Tm: Tunicamycin
UPR: Unfolded protein response
α-SMA: Alpha smooth muscle actin
