# Supplementary material for: Promoting ER stress in a plasmacytoid dendritic cell line drives fibroblast activation
Source: Cell Commun Signal. 2025 Feb 7;23:66. doi: 10.1186/s12964-025-02057-7 (PMC11804055; doi:10.1186/s12964-025-02057-7)
Supplement: Supplementary file 1 — Supplementary Material 1 [file 12964_2025_2057_MOESM1_ESM.docx]

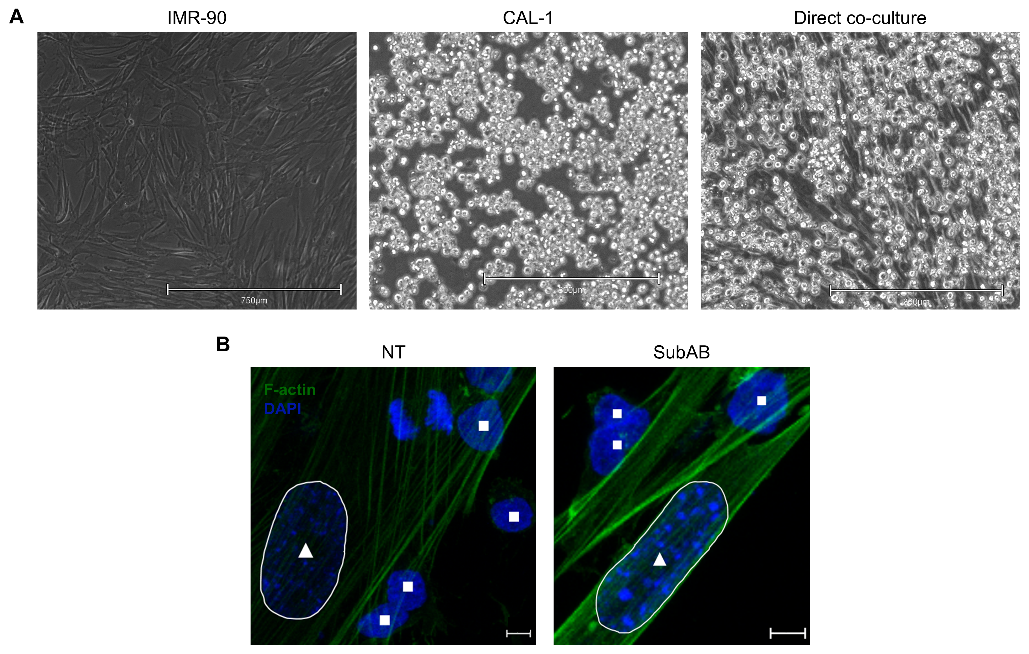


**Figure S1. Identification of IMR-90 and CAL-1 by microscopy. (A)** Brightfield images of IMR-90 and CAL-1 monocultures and in co-culture. (B) Non-treated and SubAB-treated co-cultures (1 h) were extensively washed to remove non-adherent cells, and stained for F-actin (green) and with DAPI (blue). Nuclei of IMR-90 and CAL-1 are identified with a triangle and a square, respectively. The borders of IMR-90 nuclei are limited by a white line. Scale bar = 5 µm.


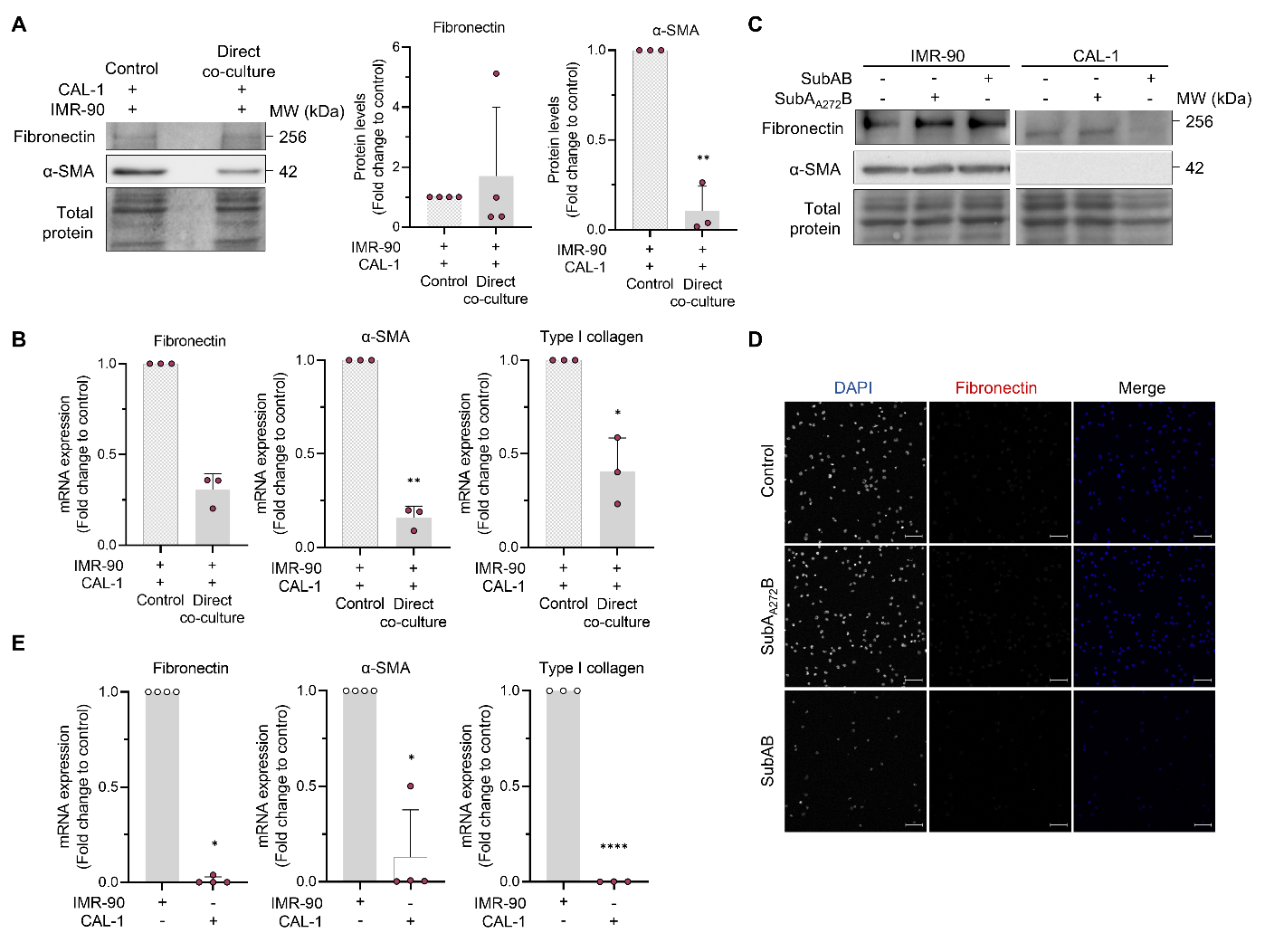


**Figure S2. Fibronectin and α-SMA expression in IMR-90 and CAL-1 monocultures and co-cultures.** Cells were either co-cultured together in a 1:10 ratio (IMR-90:CAL-1; direct co-culture), or cultured separately, and then pellets were combined (control) for a controlled analysis of fibronectin and α-SMA **(A)** protein (72 h) and **(B)** mRNA levels (24 h). **(C-E)** IMR-90 and/or CAL-1 cells were cultured alone and **(C, D)** protein (24 h and 48 h, respectively) and **(E)** mRNA levels (24 h) of fibronectin and α-SMA were analysed. Images shown in (D) are representative of 3 biological replicates (scale bar = 50 μm). Fibronectin is presented in red and DAPI in blue. In (E) mRNA levels of type I collagen were also assessed. Data represents mean ± SD and each dot represents a biological replicate. Representative blots of at least 3 biological replicates, except CAL-1 in (C) that was performed twice. Statistical significance was evaluated using Mann-Whitney test or unpaired t test with Welch’s correction accordingly to data distribution.


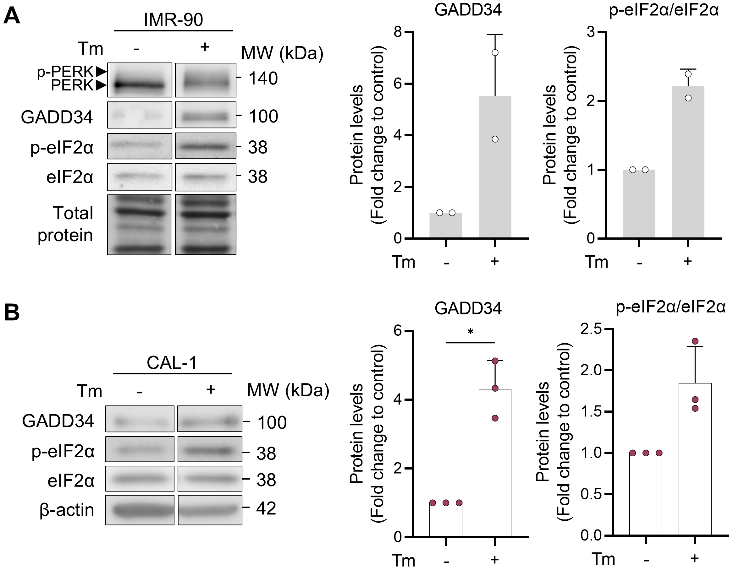


**Figure S3. Tm induces ER stress in IMR-90 and CAL-1.** **(A)** IMR-90 and **(B)** CAL-1 cells were treated with Tm for 6 h and expression of PERK, GADD34, and phosphorylated eIF2α was analysed by immunoblot. Upon activation, an upper band appears, corresponding to p-PERK. Data represents mean ± SD and each dot represents a biological replicate.


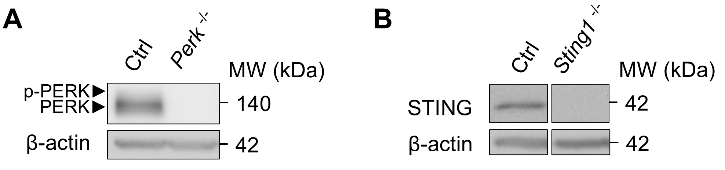


**Figure S4. Description of *Perk*^-/-^ and *Sting1*^-/-^ CAL-1 cells**. **(A)** PERK and **(B)** STING protein levels in *Perk*^-/-^ and *Sting1*^-/-^ CAL-1 cells were analysed by Western blotting.
