# Supplementary material for: Promoting ER stress in a plasmacytoid dendritic cell line drives fibroblast activation
Source: Cell Commun Signal. 2025 Feb 7;23:66. doi: 10.1186/s12964-025-02057-7 (PMC11804055; doi:10.1186/s12964-025-02057-7)
Supplement: Supplementary file 2 — Supplementary Material 2 [file 12964_2025_2057_MOESM2_ESM.pptx]

## Slide 1
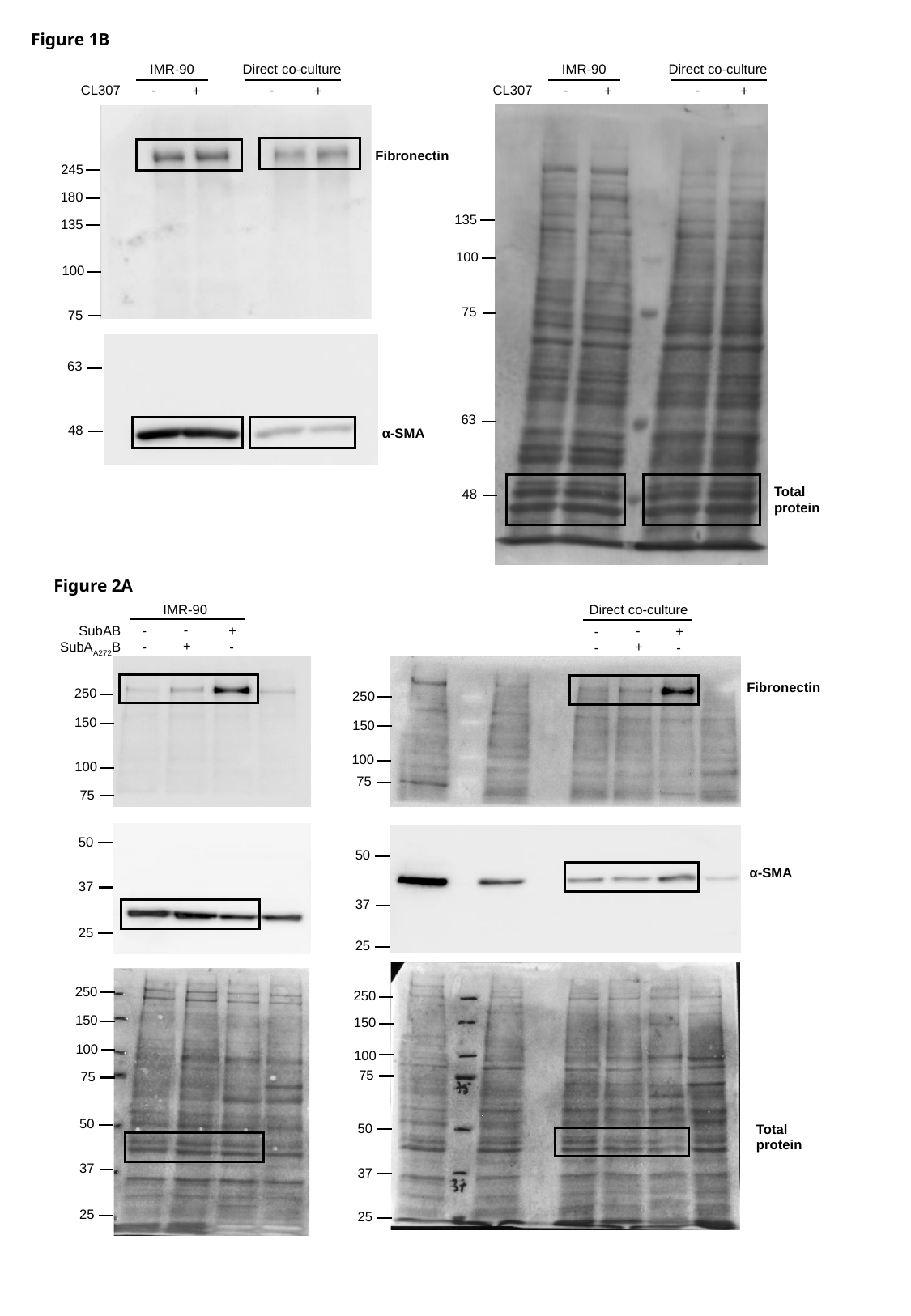

Figure 1B
IMR-90
Direct co-culture
IMR-90
Direct co-culture
-
-
-
-
CL307
CL307
+
+
+
+
Fibronectin
245
180
135
135
100
100
75
75
63
63
48
α-SMA
Total protein
48
Figure 2A
IMR-90
Direct co-culture
-
+
-
-
+-
-
+
SubAB
SubAA272B
-
-
+-
Fibronectin
250
250
150
150
100
100
75
75
50
50
α-SMA
37
37
25
25
250
250
150
150
100
100
75
75
50
50
Total protein
37
37
25
25

## Slide 2
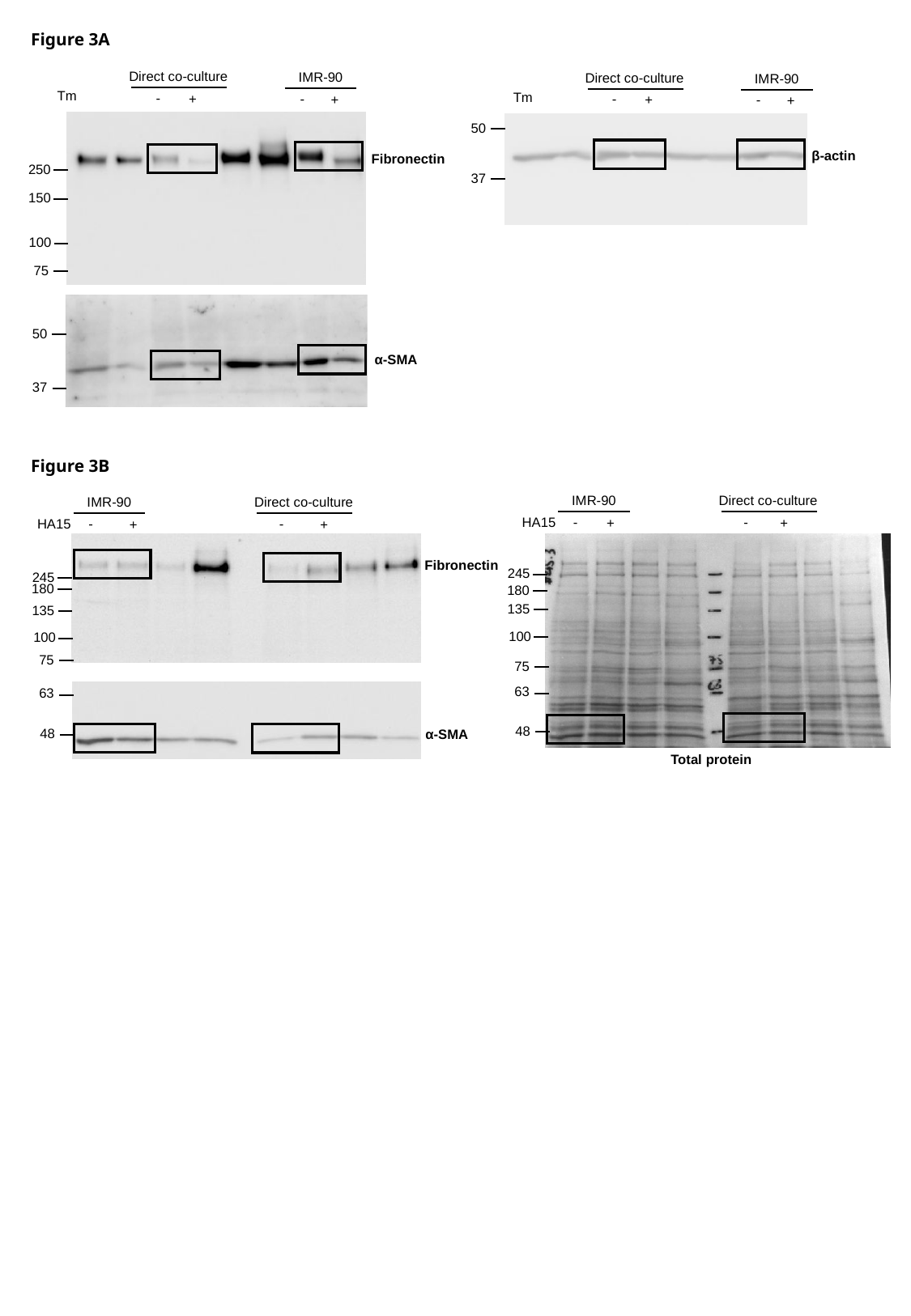

Figure 3A
Direct co-culture
IMR-90
Direct co-culture
IMR-90
Tm
Tm
-
+
-
-
+
+
-
+
50
β-actin
Fibronectin
250
37
150
100
75
50
α-SMA
37
Figure 3B
IMR-90
Direct co-culture
IMR-90
Direct co-culture
-
-
HA15
+
+
-
-
HA15
+
+
Fibronectin
245
245
180
180
135
135
100
100
75
75
63
63
48
48
α-SMA
Total protein

## Slide 3
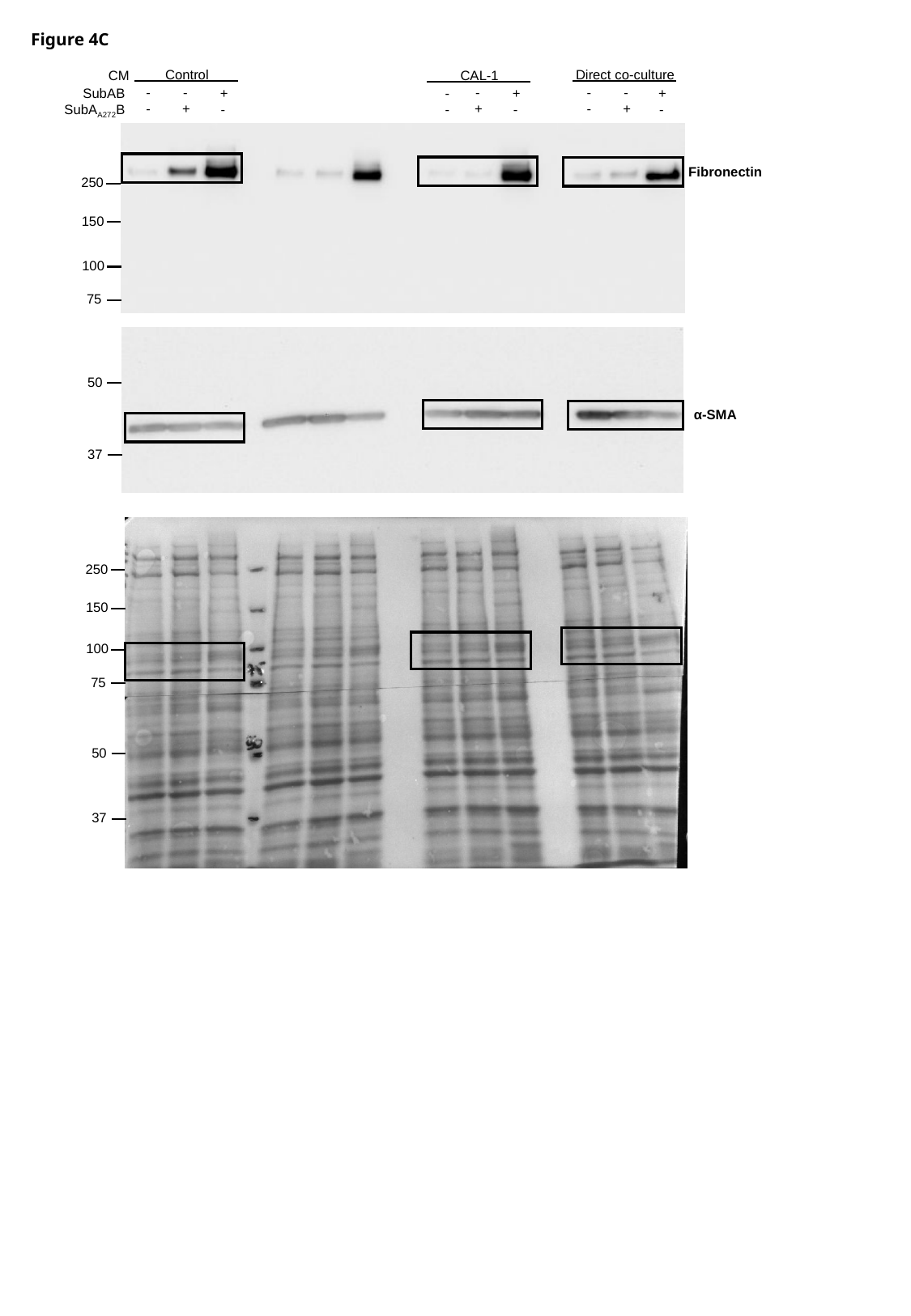

Figure 4C
Control
Direct co-culture
CM
CAL-1
-
+
-
+
-
-
-
-
-
+
+-
+-
SubAB
SubAA272B
-
-
+-
Fibronectin
250
150
100
75
50
α-SMA
37
250
150
100
75
50
37

## Slide 4
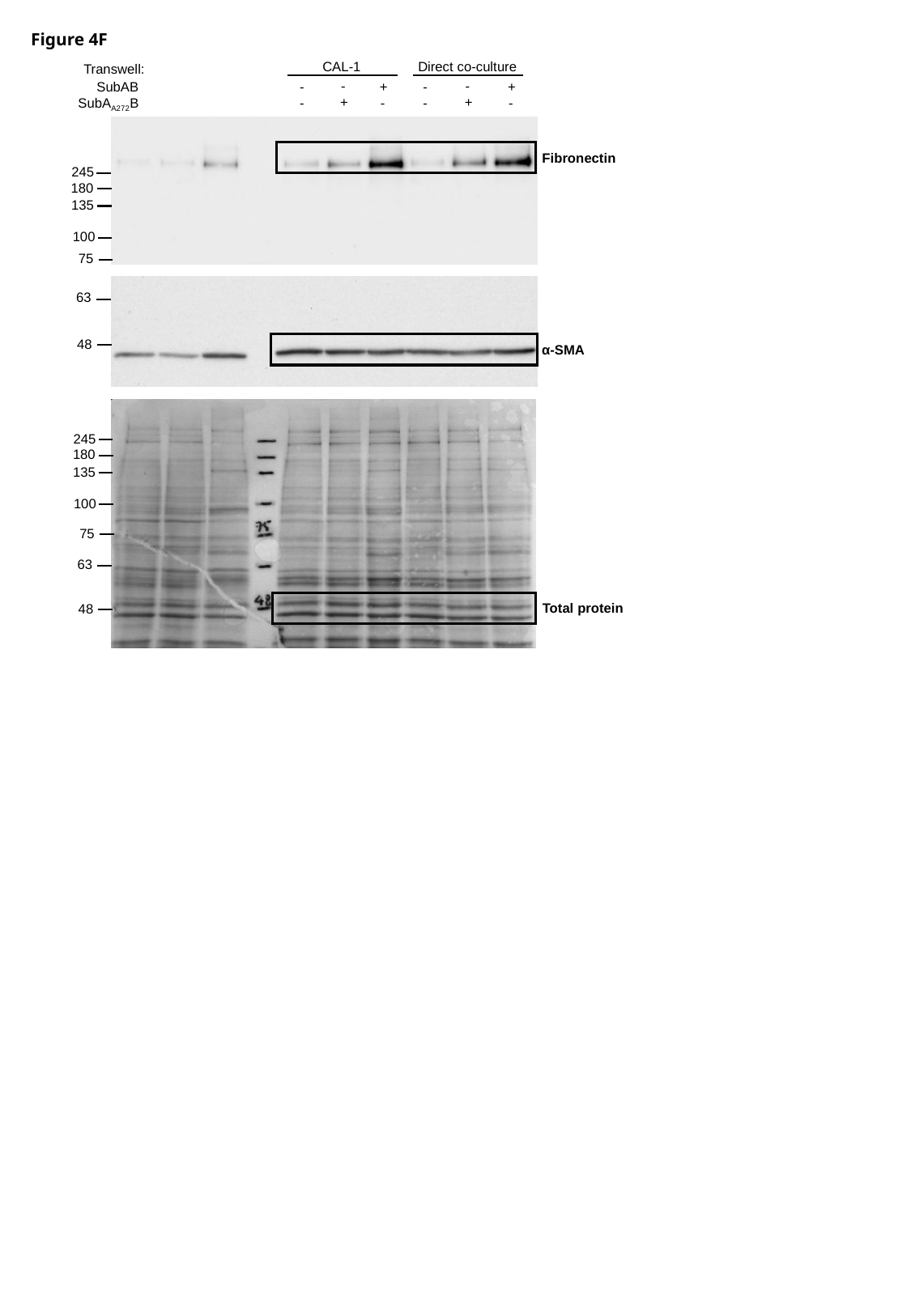

Figure 4F
CAL-1
Direct co-culture
Transwell:
-
+
-
+
-
-
-
-
+-
SubAB
SubAA272B
+-
Fibronectin
245
180
135
100
75
63
48
α-SMA
245
180
135
100
75
63
Total protein
48

## Slide 5
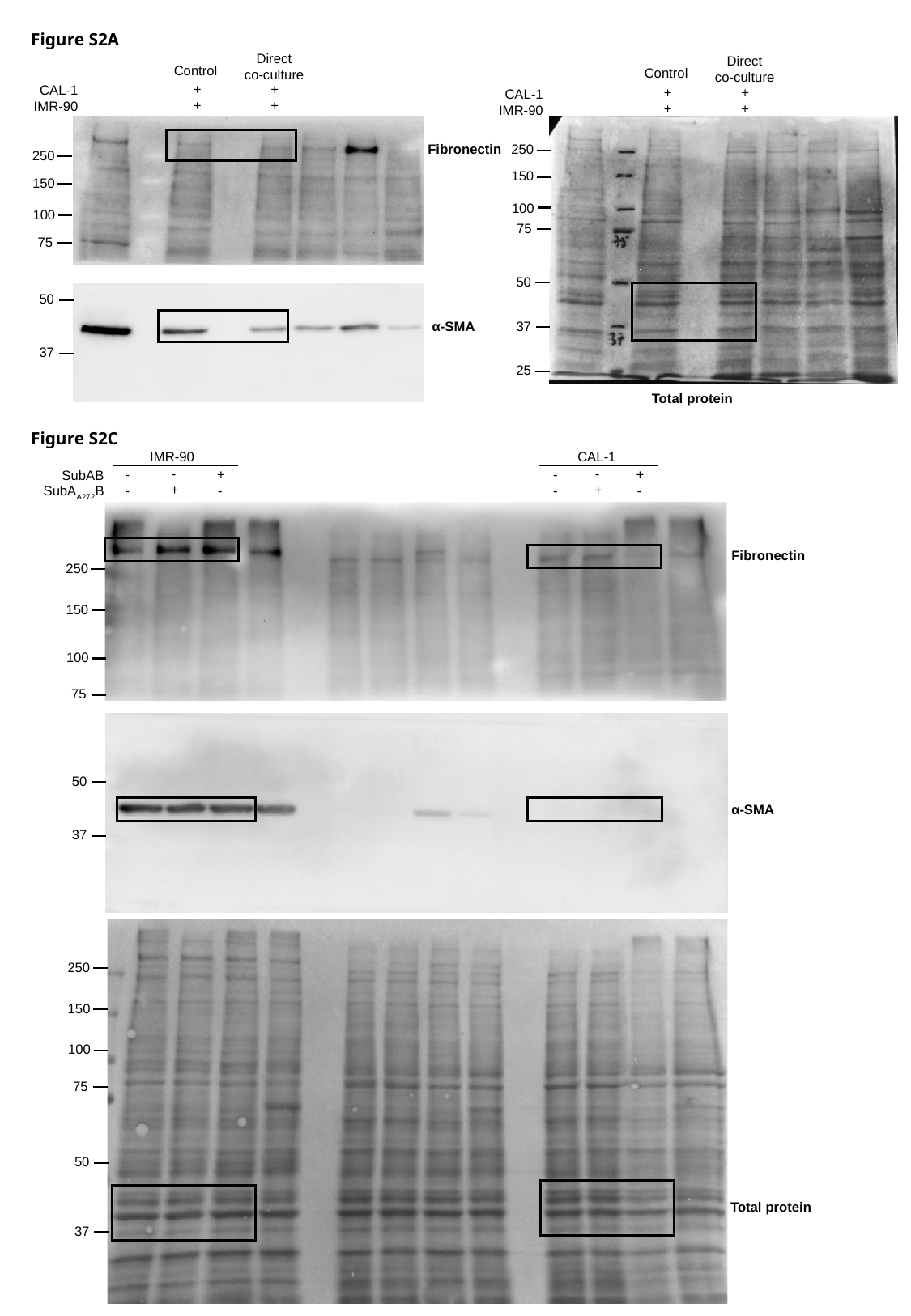

Figure S2A
Direct
co-culture
Direct
co-culture
Control
Control
+
+
+
+
CAL-1
IMR-90
+
+
+
+
CAL-1
IMR-90
Fibronectin
250
250
150
150
100
100
75
75
50
50
37
α-SMA
37
25
Total protein
Figure S2C
IMR-90
CAL-1
-
+
-
+
-
-
-
-
+-
+-
SubAB
SubAA272B
Fibronectin
250
150
100
75
50
α-SMA
37
250
150
100
75
50
Total protein
37

## Slide 6
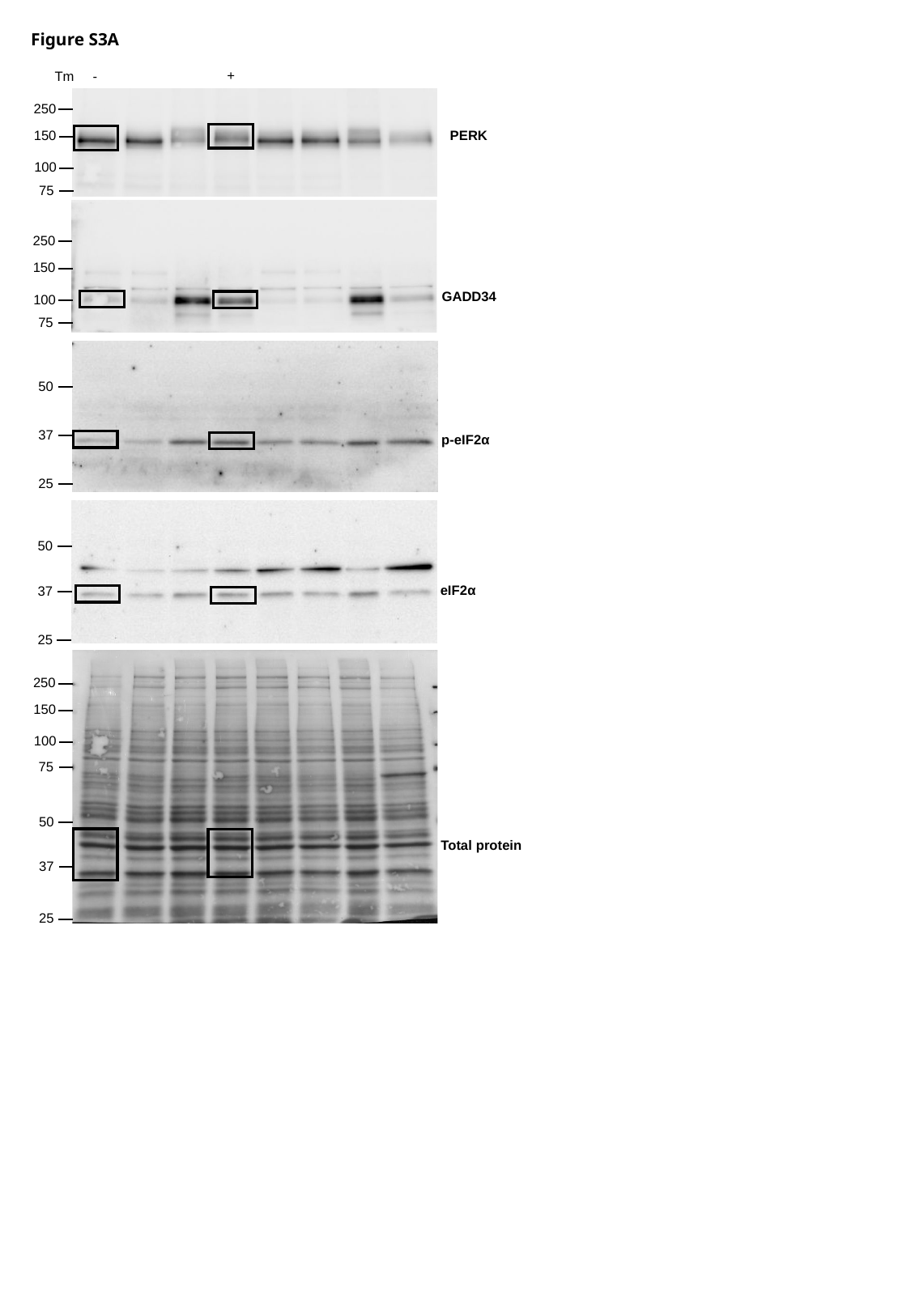

Figure S3A
+
-
Tm
250
150
PERK
100
75
250
150
GADD34
100
75
50
37
p-eIF2α
25
50
eIF2α
37
25
250
150
100
75
50
Total protein
37
25

## Slide 7
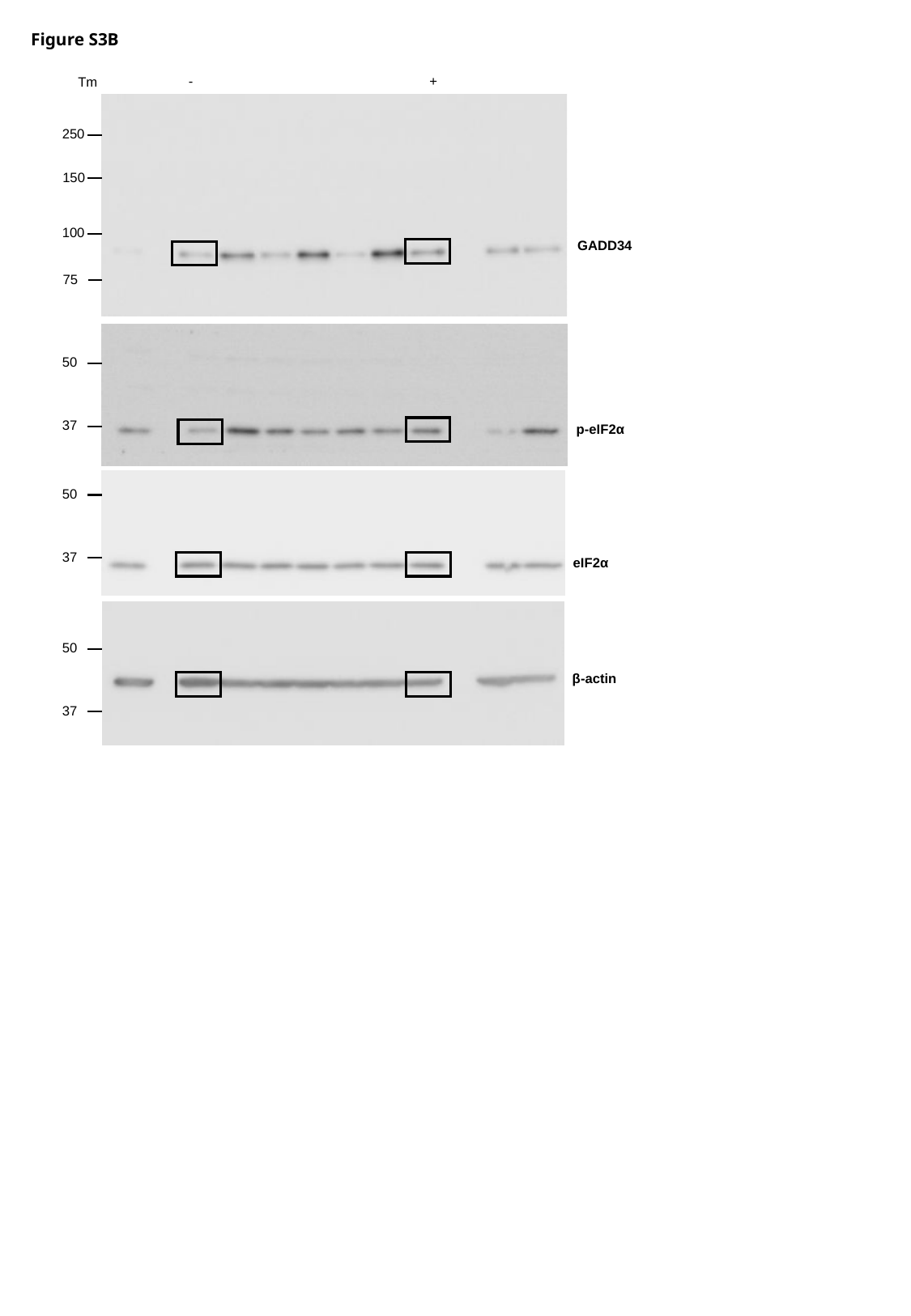

Figure S3B
+
-
Tm
250
150
100
GADD34
75
50
37
p-eIF2α
50
37
eIF2α
50
β-actin
37

## Slide 8
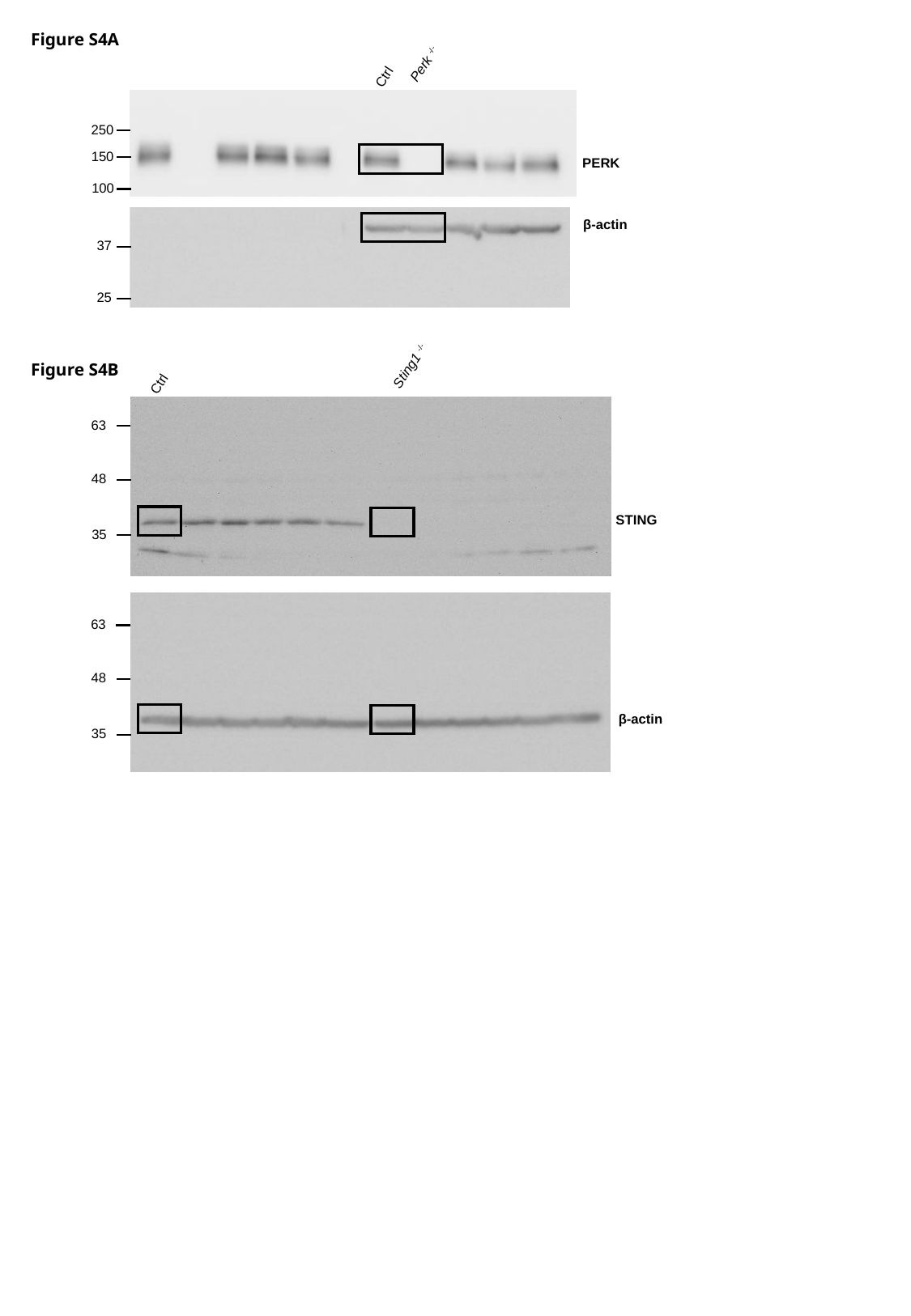

Figure S4A
Perk -/-
Ctrl
250
150
PERK
100
β-actin
37
25
Sting1 -/-
Figure S4B
Ctrl
63
48
STING
35
63
48
β-actin
35
